# dnAQET: a framework to compute a consolidated metric for benchmarking quality of de novo assemblies

**DOI:** 10.1186/s12864-019-6070-x

**Published:** 2019-09-11

**Authors:** Gokhan Yavas, Huixiao Hong, Wenming Xiao

**Affiliations:** 1 Bioinformatics Branch, Division of Bioinformatics and Biostatistics, National Center for Toxicological Center, U.S Food and Drug Administration, Jefferson, AR 72079 USA; 2 Present Address: Molecular Pathology Cytology Branch, Division of Molecular Genetics and Pathology, Office of In Vitro Diagnostics and Radiological Health, the Center for Devices and Radiological Health, U.S Food and Drug Administration, Silver Spring, MD 20993 USA

**Keywords:** de novo genome assembly, Assembly quality assessment, Next generation sequencing, Misassembly

## Abstract

**Background:**

Accurate de novo genome assembly has become reality with the advancements in sequencing technology. With the ever-increasing number of de novo genome assembly tools, assessing the quality of assemblies has become of great importance in genome research. Although many quality metrics have been proposed and software tools for calculating those metrics have been developed, the existing tools do not produce a unified measure to reflect the overall quality of an assembly.

**Results:**

To address this issue, we developed the de novo Assembly Quality Evaluation Tool (dnAQET) that generates a unified metric for benchmarking the quality assessment of assemblies. Our framework first calculates individual quality scores for the scaffolds/contigs of an assembly by aligning them to a reference genome. Next, it computes a quality score for the assembly using its overall reference genome coverage, the quality score distribution of its scaffolds and the redundancy identified in it. Using synthetic assemblies randomly generated from the latest human genome build, various builds of the reference genomes for five organisms and six de novo assemblies for sample NA24385, we tested dnAQET to assess its capability for benchmarking quality evaluation of genome assemblies. For synthetic data, our quality score increased with decreasing number of misassemblies and redundancy and increasing average contig length and coverage, as expected. For genome builds, dnAQET quality score calculated for a more recent reference genome was better than the score for an older version. To compare with some of the most frequently used measures, 13 other quality measures were calculated. The quality score from dnAQET was found to be better than all other measures in terms of consistency with the known quality of the reference genomes, indicating that dnAQET is reliable for benchmarking quality assessment of de novo genome assemblies.

**Conclusions:**

The dnAQET is a scalable framework designed to evaluate a de novo genome assembly based on the aggregated quality of its scaffolds (or contigs). Our results demonstrated that dnAQET quality score is reliable for benchmarking quality assessment of genome assemblies. The dnQAET can help researchers to identify the most suitable assembly tools and to select high quality assemblies generated.

**Electronic supplementary material:**

The online version of this article (10.1186/s12864-019-6070-x) contains supplementary material, which is available to authorized users.

## Background

With the introduction of Next Generation Sequencing (NGS) technology, it is now possible to generate massive amounts of genome sequencing data, which has enabled the de novo assembly of genomes of the sequenced individuals [1]. Approximately a decade ago, NGS technology enabled the sequencing of the genomes of two individuals, James D. Watson and Craig Venter, with relatively high coverage and generation of their diploid de novo genome assemblies [2, 3]. This was followed by the diploid de novo genome assemblies of an Asian individual genome (YH), a Korean individual (AK1) and sample NA18507, which were reported and compared to the previously available individual genomes [4–6]. More recently, diploid DNA sequences for seven individuals were assembled and reported along with the software pipeline developed for the assembly [7]. As sequencing has become cheaper and more affordable, the challenge of routinely applying NGS in the precision medicine era largely rests on bioinformatics solutions, especially for personal genome assembly in near future. For this purpose, various assembly tools have been proposed and reported in the literature for de novo assembly using short-read and long-read NGS data. Some examples of such tools are SOAPDenovo2 [8], ALLPATHS-LG [9], ABySS [10], MaSuRCA [11] and SPAdes [12], which use the short-read data along with long range mate-pair libraries, Canu [13], MECAT [14], Celera [15] and Falcon [16], which utilize long-read data produced by platforms such as Oxford Nanopore and PacBio to generate de novo assemblies. As the approaches utilized in these assembly tools differ, the quality of the scaffolds or contigs (contiguous sequences) and the assemblies produced by them also varies significantly. To address the problem of comparing these assembly software, efforts were spent by the Assemblathon consortium to evaluate the performances of multiple assemblers on simulated (artificial) data [17] and data from non-mammalian vertebrate species [18]. Despite no package was singled out as the best solution for genome assembly, these two studies established some key measurements for overall quality of an assembly. In another study [19], the authors compared de novo assemblies generated by multiple assembly tools for human chromosome 14 and three other organisms with small genomes. These works established the groundwork for some of the well-accepted metrics to measure the quality of an assembly from multiple perspectives.

A handful of tools have been reported in the literature for evaluating the quality of the newly assembled genomes using these metrics. REAPR [20] is a reference-free tool that uses the approach of aligning the reads used for the assembly back to the assembled contigs to generate quality metrics. On the other hand, QUAST [21] and its improved version QUAST-LG [22] are tools that can evaluate de novo genome assemblies in the presence of a trusted reference genome. These tools align contigs or scaffolds of a de novo assembly to the chromosomes of a given refence genome and report the quality metrics based on these alignments. They also report some reference-free metrics such as *N50* value, but the main purpose of these tools is to utilize a reference genome to generate metrics such as *genome coverage ratio*, *number of misassemblies in the contigs* etc., which are dependent on the reference genome. The main shortcomings of them are that the quality of individual contigs are not fully assessed and they do not generate a single quality metric that can reflect the overall quality of a de novo genome assembly. The multiple metrics reported by these tools may contradict each other, which can confuse the end-user when comparing and ranking multiple assemblies. It is also possible that these metrics may perform inconsistently across multiple assemblies for ranking, which makes them less reliable for ranking the assemblies in terms of their overall qualities.

To remedy this problem, we developed the **d**e **n**ovo **A**ssembly **Q**uality **E**valuation **T**ool (dnAQET) that assesses the quality of a de novo assembly using the quality scores of its individual scaffolds/contigs via consolidation of multiple well-established metrics. To demonstrate the effectiveness of the quality score generated by dnAQET, we applied our tool to four different synthetically created assembly sets, each of which was designed to evaluate one aspect of our quality score formulation. The first dataset demonstrated that dnAQET’s quality scores were better for assemblies with larger scaffolds. Using the second dataset, we showed that as the number of misassemblies identified in the scaffolds of the assemblies increased, the quality scores we assigned to these low-quality (i.e., with more misassembly) assemblies decreased. The third dataset was designed to present that the increasing reference genome coverage of the assemblies had a positive impact on the dnAQET’s quality score computed for those assemblies, which was concordant with the expectation. Finally, using the fourth dataset we investigated how the increasing redundancy in the assemblies resulted in lower dnAQET scores assigned to them.

We also computed the individual quality scores of chromosomes as well as the overall quality scores for available reference genome builds for five organisms (four mammalian and one fish) and showed that the quality assessment of dnAQET for these reference builds was concordant with the expectation that a more recent build of a reference genome should be better assembled than an older one. Furthermore, we showed that the well-established metrics could present contradictory results to each other using six de novo assemblies for sample NA24385 and our quality score was effective to unify these metrics into a single score to reflect their overall quality. It is also important to note that the top performing metrics when ranking reference genome builds did not perform well for ranking six NA24385 de novo assemblies. On the other hand, dnAQET’s overall quality score was very consistent in ranking the assemblies in both datasets, which proved its reliability for assessing the assembly quality and its suitability to be used as a benchmarking metric to compare de novo assemblies. We also showed that dnAQET is very fast, scalable and capable of handling assemblies for large and complex genomes such as human using a reasonable amount of computational memory.

## Results

The dnAQET framework comprises of two main steps: (i) aligning assembled scaffolds (contigs) to a trusted reference genome and then (ii) calculating quality scores for the scaffolds and the whole assembly. For the alignment step, dnAQET provides two separate alignment tools for users to choose. The first one is the Nucmer pipeline, from the MUMmer4 package [23], whose predecessor in MUMmer3 package [24] is a very widely used general purpose alignment tool to map long DNA sequences. The more recently released MUMmer4 package contains a much faster and memory efficient Nucmer version that can handle large genomes. The most recent version of the Nucmer is used in dnAQET. The second option for alignment that dnAQET offers is the Minimap2 aligner [25], which is also a very fast pairwise aligner for nucleotide sequences.

To enhance the computational performance, the alignment process is broken down into three sub-steps: (a) partitioning of the reference genome and the assembly files into smaller chunks, (b) aligning each partition of the assembly against each partition of the reference genome in parallel, and finally (c) filtering the redundant and overlapping alignments for each scaffold. After filtering, the remaining alignments are used to compute the quality scores.

### Scalable alignment of scaffolds to a reference genome

The dnAQET handles alignment of scaffolds to a reference genome in a parallelized manner by partitioning the assembly file into multiple approximately-equal sized files and the reference genome into multiple reference files, each containing a single chromosome (Additional file 1: Figure S1), enabling the method to be scalable for handling assemblies of large genomes. The total number of partition files of an assembly can be determined by the user, but its default value is set to one. The tool distributes the scaffolds to the user specified number of files in such a way that the total number of base pairs contained in each file would be similar across the partitions. For partitioning the genome, dnAQET distributes the chromosomes of the reference genome into multiple files so that each assembly partition can be aligned to a single chromosome independently on a High-Performance Computing (HPC) or a multi-threaded computing environment.

### Computing quality scores for scaffolds

The dnAQET parses the alignment results for each scaffold and filters out the redundant and ambiguous alignments to obtain the longest consistent matches between the scaffold and the reference. For this purpose, we adapted the underlying algorithm of *delta-filter* utility in the MUMmer package and implemented the same approach in dnAQET. When the alignment step is completed either using Nucmer or Minimap2, the alignment results are scanned using appropriate parsers specifically designed to parse data in the corresponding alignment format. They are then converted into an internal alignment format to be filtered using our filtering algorithm. Note that the filtering step is independent of the chosen alignment tool and is applied to all alignment results no matter what tool is used to generate them. We then compute a quality score of an individual contig using the set of best alignments based on the total number of aligned base pairs in the scaffold (*reward*), the total number of misassembly determined in the scaffold (*penalty*), and the length of the scaffold (*length scaling coefficient*).

The reward of a scaffold is supposed to be directly proportional to the total number of bases that are aligned to the reference genome by the alignment tool. Based on this assumption, dnAQET assigns a reward value, denoted by *R*(s), to a scaffold *s* that is equivalent to the ratio of the total number of aligned bases to the total number of bases in *s* without the scaffolding gaps.

$$ R(s)=\frac{Total\ number\ of\ aligned\ base\ pairs\ in\ s}{Total\ number\ of\ base\ pairs\ in\ s- Total\ number\ of\ base\ pairs\ in\ the\ scaffolding\ gaps} $$

In the above formulation, the total number of ambiguous base pairs, which are detected to be scaffolding gaps, are subtracted from the total size of the scaffold (see the denominator in the above formula). This avoids improper reduction of the reward of a scaffold.

**Fig. 1:**
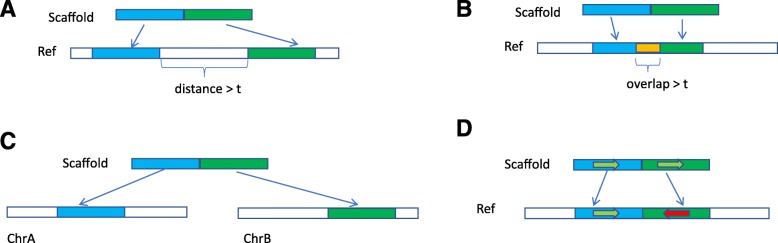
Illustration of misassembly types. The green and blue bars indicate two flanking sequences of a scaffold and the white bars represent regions in a reference genome. The relocations with a distance (**a**) and an overlap (**b**) are marked with white and yellow bars under the reference bars, respectively. Translocation is denoted by two chromosomes (**c**). The arrows depict the directions of strands with red to show the inversion (**d**)

The penalty assigned to a scaffold is directly related to the misassembly, which is basically summarized as the inconsistencies between the flanking alignments of a scaffold and the reference. In dnAQET, we consider three types of misassembly, which is also consistent with the types and definitions of the misassembly reported by previous literature [21]. These misassembly types (Fig. 1) are described in detail below.

Relocation, a type of misassembly in a scaffold, can happen in two cases: two consecutive sequence segments of the scaffold are aligned on the same chromosome with a separation distance of more than *t* base pairs (Fig. 1a), or they are aligned on the same chromosome with an overlap of more than *t* base pairs (Fig. 1b). The relocation threshold *t* is 1000 base pairs by default and can be adjusted by the user. A distance or overlap between the alignments of the flanking sequences smaller than *t* is not considered as a relocation. Translocation is a type of misassembly that is observed when the two flanking sequences of a scaffold are aligned to two different chromosomes in the reference genome (Fig. 1c). Inversion is a type of misassembly that occurs in cases where the two flanking sequences of a scaffold are aligned in the opposite strands of the same chromosome (Fig. 1d).

In theory, one would expect a perfect alignment of each reference chromosome (scaffold) back to itself. However, due to gaps (ambiguous sequences), repetitive sequences in the reference genome and limitations of alignment algorithms, it is still possible to observe misalignment between two identical long fragment sequences (> 1 MB), which is considered as artifact of MUMmer and MiniMap2. Thus, dnAQET considers the fact that some artifacts should be expected even though a scaffold is assembled perfectly, and these artifacts should be correlated to the length of assembled scaffold, i.e. the longer a scaffold is, the more artifacts should be expected.

When dnAQET processes a scaffold for computing the penalty, it needs to decide how the misassembly detected in this scaffold compares to the artifact expected from a scaffold of this size (*l*) with a given relocation threshold (*t*). The dnAQET uses a regression model to find the expected artifact given a scaffold with size, *l*, and a relocation threshold, *t*, which is used to decide the relocation type of misassembly. For each reference chromosome, dnAQET first randomly creates a set of artificial contigs/scaffolds, which cover the whole chromosome with 1X depth of coverage. Then these contigs, along with the original reference chromosome sequence, are aligned back to the whole reference genome. At last, the total number of misassembly is computed for each contig/scaffold and for each relocation threshold *t* in the range [100, 10,000] with a 100 base pair increments. The computed artifact is fit to the following model:

$$ \varepsilon ={\alpha}_cl+{\beta}_ct+{k}_c $$

via least-squares regression, where *ε* is the artifact; *α*_c_ and *β*_c_ are model parameters and *k*_c_ is the intercept for the model obtained for chromosome *c*. To determine the model parameters and the intercept, dnAQET fits the observed artifact in each of the chromosomes (scaffolds) of the given reference genome in the above model. Additional file 2: Tables S1–S5 provide the computed values for these coefficients for the chromosomes of the latest builds of human, chimpanzee, mouse, rat and zebrafish genome assemblies from University of California, Santa Cruz (UCSC) Genome Browser web site [26].

After all model parameters for each chromosome in the reference are identified, dnAQET computes the expected misassembly, *ε*_s_, for a scaffold *s* as follows:

$$ {\varepsilon}_s=\left\{\begin{array}{c}{\alpha}_c{l}_s+{\beta}_c{t}_u+{k}_c if\ \left({\alpha}_c{l}_s+{\beta}_c{t}_u+{k}_c\right)>0\\ {}0 otherwise\end{array}\right. $$

where *l*_s_ denotes the length of the scaffold, *t*_u_ is the relocation threshold set by the user, *α*_c_, *β*_c_ and *k*_c_ are the model coefficients computed for chromosome *c*, which contains most of the alignments for scaffold *s*. Finally, the penalty assigned to scaffold *s* by dnAQET, denoted by *P*(*s*), is computed as follows:

$$ P(s)=\left\{\begin{array}{c}{\mathit{\log}}_{100}\ \left({m}_s-{\varepsilon}_s\right) if\ \left({m}_s-{\varepsilon}_s\right)>1\ \\ {}0 otherwise\end{array}\right. $$

where *m*_*s*_ represents the total number of misassembly that dnAQET detects in the scaffold *s*. When the difference between the observed misassembly (*m*_s_) and the expected artifact (*ε*_s_) for this scaffold with the specified relocation threshold is less than or equal to one, there is no penalty assigned to this scaffold. Otherwise, the logarithmic value of this difference in the base of 100 is used as the penalty to scaffold *s*.

The length of a scaffold is an important indication of assembly quality. A well-assembled and high-quality scaffold should be equal to or longer than the shortest chromosome of the trusted reference genome, against which the quality is computed. The length of the shortest chromosome of reference genome *G*, is called length scaling factor of *G* and denoted by *θ*_G_. It is used as a benchmarking value to assess the quality of a scaffold of the de novo assembly. Thus, dnAQET incorporates a coefficient called length scaling coefficient of a scaffold *s* in its quality score calculation formulation, which is denoted by *L*(*s*). This coefficient is computed with respect to the shortest reference chromosome length (*θ*_G_) using the following equation:

$$ L(s)=\left\{\begin{array}{c}1 if\ {l}_s\ge {\uptheta}_G\\ {}\frac{-1}{\log_{10}\left(\frac{l_s}{\uptheta_G}\right)-1}\  otherwise\end{array}\right. $$

When the scaffold is larger than or equal to the shortest chromosome of the reference, the length scaling coefficient is set to 1. This guarantees that the contigs or scaffolds longer than the shortest chromosome are not punished. For a scaffold shorter than the shortest chromosome, the coefficient value increases as the scaffold gets longer, finally reaching one when *l*_s_ is equal to *θ*_G_. Instead of taking a simple ratio of the length of the scaffold to the length of the shortest chromosome, dnAQET uses the above function not to penalize the small to medium sized scaffolds very harshly.

After reward, penalty and length scaling coefficient are calculated, dnAQET integrates them in an overall quality score for scaffold *s*, *Q*(*s*), using below equation.

$$ Q(s)=L(s)\frac{R(s)}{1+P(s)} $$

In case dnAQET identifies no misassembly in a scaffold or the misassembly is negligible, the quality score is basically equal to the alignment ratio of this scaffold multiplied with its length scaling coefficient. On the other hand, the quality of a scaffold decreases with the increasing number of misassembly detected in the scaffold.

### Computing quality score for assembly

After quality scores for individual scaffolds (or contigs) are calculated, dnAQET computes a quality score for the whole de novo assembly using the individual quality scores and the redundancy observed in the assembly. There are three factors that dnAQET considers for quality score computation: (a) distribution of the quality scores of scaffolds of an assembly, (b) the reference genome coverage provided by the scaffolds at different quality thresholds, and (c) the redundancy of the scaffolds in the assembly.

The dnAQET framework uses the quality score distribution of the scaffolds of a de novo assembly, as a component of the final quality score. To utilize this information, the ratio of the total number of base pairs of the scaffolds having quality scores higher than certain quality thresholds to the whole assembly size is first plotted at the corresponding quality thresholds (from 0 to 1 with 0.01 increments) as a curve (see Additional file 1: Figure S2A for an example). The area under this curve is then calculated, which is denoted by *Δ*_A_, where *A* represents the assembly. In the ideal case where all scaffolds of an assembly have a perfect quality score of 1, the area under this curve is equal to one (i.e., *Δ*_A_ = 1). As it is not always possible to obtain perfectly scored scaffolds in practice, the curve may reach the maximum ratio of 1 at a lower quality threshold in most cases. In such cases, *Δ*_A_ would be much lower than one. In this respect, *Δ*_A_ value directly reflects the quality distribution of the scaffolds of a de novo assembly.

Like the cumulative scaffold quality distribution graph, we plot the cumulative reference genome coverages of the scaffolds that meet certain quality thresholds. This curve presents us crucial information from two perspectives: 1) how much reference genome coverage can be reached with the scaffolds/contigs of the assembly and 2) how the reference coverage changes with quality distribution of the scaffolds of this assembly. An example of cumulative reference genome coverage graph is given in Additional file 1: Figure S2B.

The dnAQET framework uses the area under the cumulative genome coverage ratio curve, which is denoted by *Ω*_A_ for the assembly *A*, as another component of the overall quality score function to evaluate the assembly. In case the whole reference genome is fully covered by perfect scaffolds (i.e., scaffolds with a quality score of 1) of the de novo assembly, the *Ω*_A_ is equal to 1. When the coverage is less than 100% or a high coverage is achieved with low quality scaffolds, dnAQET would reflect these less than ideal cases to the overall quality score of the assembly, by assigning a much lower value to *Ω*_*A*_.

In summary, we incorporate *Ω*_A_ to our quality score scheme to distinguish two assemblies, say *A*_1_ and *A*_2_, where both assemblies cover the same amount of the reference genome with different quality scaffolds. In such a case, the one with high quality scaffolds will have a larger area under the cumulative genome coverage curve, hence a higher *Ω*_A_ value. Thus, this component is incorporated into the final quality score formulation and is used to distinguish *A*_1_ and *A*_2_ in terms of their quality.

The last component of dnAQET’s assembly quality scoring scheme is the inverted redundancy identified in an assembly. The redundancy in an assembly is defined as the unnecessarily repeatedly assembled sequence in the de novo assembly when it is compared to the reference genome. An example is given in Additional file 1: Figure S3 where two scaffolds, *s*_1_ and *s*_2_, are aligned to a reference genome with alignments *λ*_1_ and *λ*_2_ respectively. The total covered reference genome is denoted with *Φ*. The overlap between these alignments is denoted with *o*, which is shared by *λ*_1_ and *λ*_2_ redundantly. Then the total inverted redundancy ratio of a de novo assembly, *A*, with set of scaffolds, *A* = {*s*_1_, *s*_2_,…, *s*_i_} is denoted by *Π*_A_ and computed as:

$$ {\Pi}_A=\frac{\Phi_A}{\sum_{s_i\in A}{\lambda}_i} $$

where *λ*_i_ represents the total aligned base pairs in scaffold *i* and *Φ*_A_ represents the total covered reference genome size by all scaffolds of assembly *A*. The inverted redundancy ratio is always a value between 0 and 1 for a de novo assembly. In the optimal case, where there are no redundant alignments, the inverted redundancy would be equal to 1. As this ratio gets smaller, the redundancy of an assembly increases, which makes the assembly less desirable. Therefore, it is essential to include such a component, which measures redundancy of an assembly, in our quality score computation. For this reason, we consider the inverse of the redundancy value of an assembly *A* denoted by *Π*_A_ and incorporate it into our formula.

The dnAQET framework computes the final quality score for an assembly *A* using below equation.

$$ Q(A)=\sqrt[3]{\Delta _A{\Omega}_A{\Pi}_A} $$

The final quality score is the geometric mean of the area under the cumulative distribution curve of quality scores of scaffolds, the area under the cumulative curve of genome coverage and the inverted redundancy of the de novo assembly *A*. These three components represent three aspects of a desirable assembly: (i) scaffolds should have high individual quality scores, (ii) reference genome should be mostly covered with high quality scaffolds, and (iii) redundancy in an assembly should be minimal. Note that all these three values are defined to be a real value in [0, 1], which guarantees that the quality value obtained by taking the geometric mean would always be a real value in the same interval.

### Performance evaluation of dnAQET

To evaluate the performance of dnAQET’s quality scores and compare them with the currently well-established metrics, three types of data were used: (i) in-silico scaffold data generated from the latest human reference genome build (i.e., hg38) with various genome coverages, numbers of misassemblies per scaffold and mean scaffold lengths, (ii) various builds of whole genome assemblies of five different organisms (human, mouse, rat, chimpanzee and zebrafish) obtained from UCSC Genome Browser website [26] and (iii) six de novo assemblies for the sample with National Institute of Standards and Technology (NIST) ID HG002 and Coriell ID NA24385 from the Genome in a Bottle (GIAB) project [27].

For in-silico performance analysis, we generated four types of synthetic assembly datasets from human reference build hg38, where each of these datasets were designed to analyze a different aspect of the quality computation process as follows:

(i) **Mean scaffold length dataset**: We generated scaffolds with randomly chosen coordinates covering hg38 with 1X depth of coverage. The lengths of the scaffolds were drawn from normal distributions with mean lengths of 10^4^, 10^5^, 10^6^, 10^7^ base pairs and standard deviations of 10, 10^2^, 10^3^ and 10^4^, respectively.
(ii) **Misassembly dataset**: We randomly created assemblies that contain scaffolds with 0, 10, 20, 30, 40, 50, 100, 200, 300, 400, 500, 600, 700, 800, 900 and 1000 misassemblies per scaffold from hg38. The lengths of these scaffolds were drawn from a normal distribution with a mean of 10^7^ and standard deviation of 10^4^. Each assembly had a total genome coverage of 1X.
(iii) **Coverage dataset**: Assemblies that cover 0.1X up to 1X (with increments of 0.1X) of the hg38 build were created randomly. We didn’t induce any artificial misassemblies into the scaffolds. The lengths of these scaffolds were drawn from a normal distribution with a mean and standard deviation of 10^7^.
(iv) **Redundancy dataset**: We created assemblies that covered the hg38 once (1X), twice (2X), three times (3X), four times (4X) and finally five times (5X). There were no artificially induced misassemblies and the scaffold lengths were normally distributed with a mean value of 10^7^ and standard deviation of 10^4^.

Note that for all the above described synthetic datasets, we created five different assemblies for each value of the parameter that was to be analyzed.

For testing dnAQET on human reference data, we used fifteen builds of reference genome assemblies, more specifically hg4, hg5, hg6, hg7, hg8, hg10, hg11, hg12, hg13, hg15, hg16, hg17, hg18, hg19 and hg38. The dnAQET was used to evaluate each of these genome builds against the hg38 build. Since hg38 is the most recent reference genome, it is expected to be the best among all these builds. Note that some of the reference builds (such as hg1, hg2, hg3, hg9, hg14) are missing from our study due to the unavailability to download in UCSC Genome Browser data repository.

Similarly, there were ten available builds (mm1 to mm10) of mouse reference genome, six builds (rn1 to rn6) of rat reference genome, six builds (panTro1 to panTro6) of chimpanzee reference genome and nine builds (danRer1, danRer2, danRer3, danRer4, danRer5, danRer6, danRer7, danRer10 and danRer11) of zebrafish genome. We used the latest builds mm10, rn6, panTro6 and danRer11 as the references for mouse, rat, chimpanzee and zebrafish, respectively, against which the rest of the builds were evaluated by dnAQET. We calculated the quality scores for each chromosome of the builds and for the whole reference assemblies. Two restrictions were imposed on the datasets: (i) only the primary chromosomes of these builds were included in our analysis for the sake of simplicity and (ii) to fairly evaluate human, mouse and rat genome builds, we had to exclude Y chromosome from the analysis due to the lack of availability of this chromosome for some of the earlier builds.

When using different builds of reference genomes to evaluate dnAQET’s scores, our hypothesis is that the quality scores of the chromosomes of a more recent build of the reference genome of an organism should be better than that of an older build of the reference genome of the same organism. Moreover, the overall quality score from dnAQET for the older builds should be lower than those for the later builds for all the organisms used in this work.

To test dnAQET on real genome assemblies, we used two de novo assemblies from GIAB data repository for sample NA24385. The first assembly was created using Celera assembler [15] and is available on GIAB ftp site [28]. The second assembly was generated using Falcon [16] and is available on GIAB ftp site [29]. The other four assemblies were generated in-house using the MECAT assembler [14] on the PacBio data with four different depth of coverages namely 5X, 25X, 50X and 70X. Note that the PacBio data was produced by the GIAB consortium and was freely available on GIAB ftp site [30].

### Quality analysis of synthetic data

**Fig. 2:**
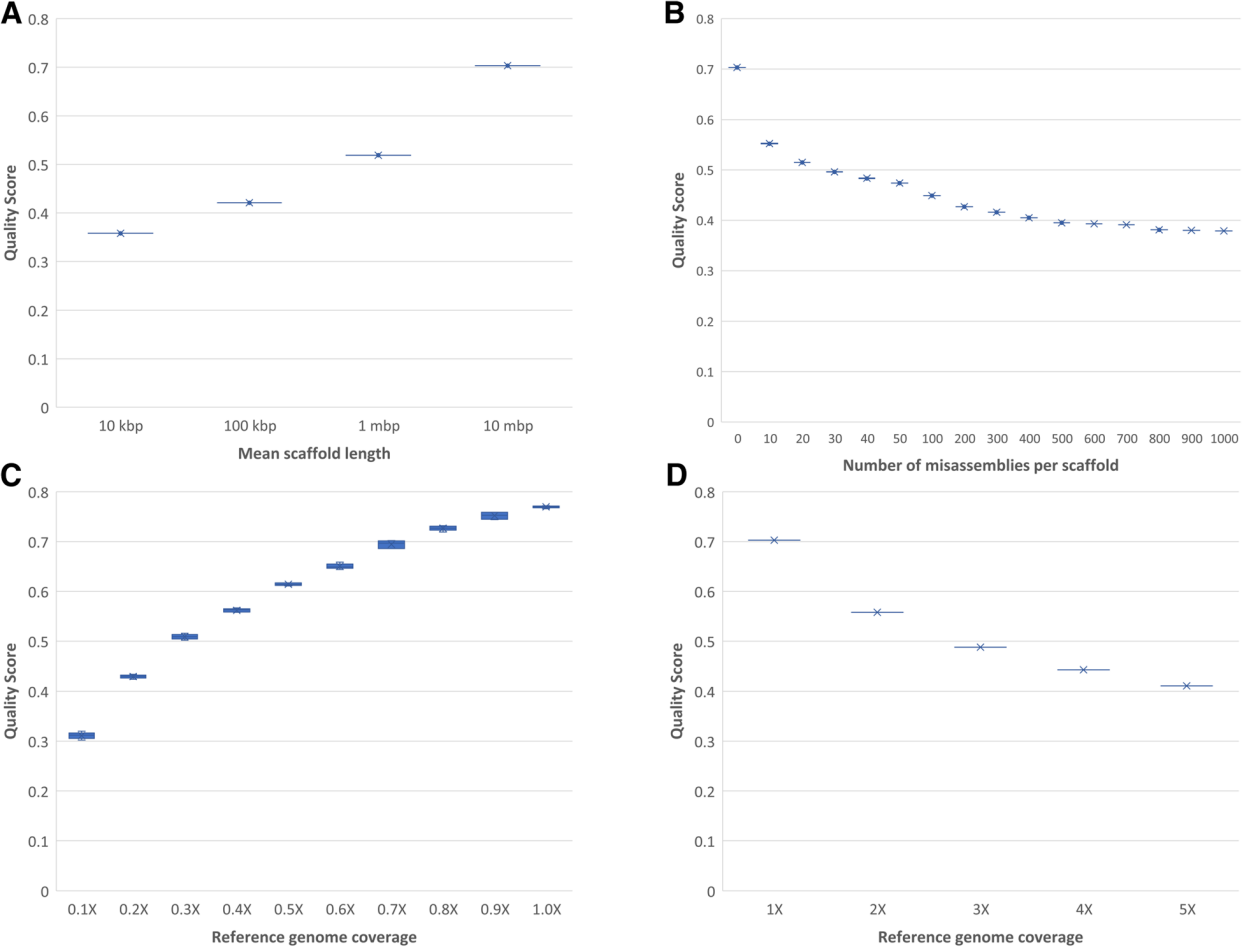
The dnAQET quality scores for synthetic datasets. The y-axis indicates the quality scores that dnAQET computed for the synthetic datasets with varying mean scaffold length (**a**), number of misassemblies per scaffold (**b**), genome coverage (**c**) and redundancy (**d**)

We first analyzed how dnAQET’s quality score changed as the mean scaffold length of the assemblies increased using the first synthetic dataset. As demonstrated in Fig. 2a, the quality scores of the assemblies increased as the average size of the scaffolds got longer. Initially, the quality scores of the assemblies with mean scaffold sizes of 10,000 base pairs concentrated around 0.35 and quality values monotonically increased for assemblies with larger scaffolds, finally becoming slightly larger than 0.7 for the assemblies with an average of 10 million base pair scaffolds. In this sense, the dnAQET’s quality score is concordant with the general assumption that an assembly with larger scaffolds should be better in quality than an assembly with shorter scaffolds given that both assemblies have same total length.

Using our second dataset, we investigated the effect of the increasing number of misassemblies in the dnAQET’s quality score formulation. As shown in Fig. 2b, the quality scores of the assemblies with no artificially induced misassembly were slightly larger than 0.7 but with the introduction of 10 misassemblies per scaffold into the assembly, we observed the quality scores dropped down to 0.55. This trend of decrease in quality scores continued with the increasing misassembly numbers, and finally quality score settled down at 0.38 for the assemblies with 1000 misassemblies per scaffold. This result is concordant with the expectation that an assembly with lower number of misassemblies should have a higher quality than the one with more misassemblies. In this respect, our quality score formulation correctly characterizes the effect of misassemblies on the quality of an assembly.

In Fig. 2c, we present the effect of increasing genome coverage in dnAQET’s quality score. As expected, the quality of an assembly with higher genome coverage was consistently higher than that of an assembly with lower coverage. The highest quality scores were obtained for assemblies with 1X coverage whereas the lowest quality scores were around 0.3 for 0.1X coverage assemblies. This result demonstrated that dnAQET’s quality score behaved as expected for changing genome coverage values.

Finally, we investigated the effect of the redundancy on our quality score formulation as presented in Fig. 2d. We computed the quality scores for datasets with 1X, 2X, 3X, 4X and 5X genome coverages, where all coverages more than 1X corresponded to redundant assemblies. As presented in Fig. 2d, the quality scores of the assemblies decreased as their redundancy increased. This result is concordant with the hypothesis that assemblies with unnecessary, repeated scaffolds should be in lower quality than concise assemblies.

Overall, these results we obtained using synthetic data reflects the capability of dnAQET’s quality score to capture and combine different aspects of quality evaluation into a single formula.

### Quality of reference genome builds of five organisms

**Fig. 3:**
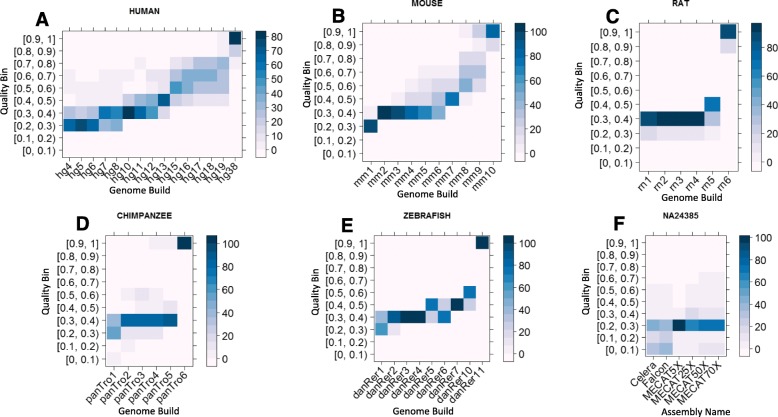
Heatmaps of quality scores of chromosomes and scaffolds for the reference genome builds of five organisms and the de novo assemblies of NA24385. The x-axis depicts the builds or assemblies. The y-axis indicates the ten quality scores bins between 0 and 1. The color given in the legend shows the percentage of the chromosomes and scaffolds that fall in the quality bin indicated by the y-axis. The quality score heatmaps are for human (**a**), mouse (**b**), rat (**c**), chimpanzee (**d**) and zebrafish (**e**) reference chromosomes from specified builds and the scaffolds of de novo assemblies for sample NA24385 (**f**) from six different assemblies. Note that there are 10 quality interval bins, dividing the [0, 1] range into nine equal sized left-closed, right-open intervals and a single closed interval, which is [0.9, 1]

The quality score distribution of chromosomes for different builds of reference genomes for the five organisms are given in Fig. 3. The segregation of reference genome builds is clear for all five organisms in terms of the quality scores of chromosomes calculated by dnAQET. For example, the quality score distributions of human reference genome builds (Fig. 3a) revealed that approximately 65% of the chromosomes in the earlier versions of human reference genome (i.e., builds hg4 to hg6) had quality scores in the range of 0.2 to 0.3. In contrast, the quality scores of the chromosomes of the newer builds hg7 to hg12 were improved to the range of 0.3 to 0.4 that included more than 48% of the chromosomes in the worst case. The shift of the quality scores towards the higher quality bins continued to reach the point where more than 50% of the chromosomes of hg13 had quality scores between 0.4 and 0.5. Majority of the chromosomes in builds hg15 and hg16 had quality scores in the range of 0.5 to 0.7 whereas the majority of the hg17, hg18 and hg19 chromosomes had quality scores between 0.6 and 0.8. Finally, the percentage of the high-quality chromosomes (i.e., chromosomes with quality scores higher than 0.8) reached to 95% for build hg38.

Similarly, we found that there was a clear segregation between the quality scores of chromosomes of the earlier and the most recent versions of the mouse reference genomes as presented in Fig. 3b. In the earlier versions, such as the builds mm2 to mm6, the quality scores of chromosomes were concentrated in the [0.3, 0.4) interval. However, majority of the chromosomes of build mm7 had quality scores in the [0.4, 0.5) bin. On the other hand, the ratio of medium to high quality chromosomes (i.e., chromosomes with a quality higher than 0.5 and less than 0.8) gradually increased for builds mm8 and mm9. Finally, more than 85% of the chromosomes of the latest build mm10 had quality scores higher than 0.9.

Overall, for the five organisms, the chromosome quality scores from dnAQET were higher for more recent builds. These results were concordant with our hypothesis and evidently demonstrated that dnAQET could be used to fairly evaluate quality of scaffolds and contigs. Additionally, we present the individual quality scores of the chromosomes for each genome build of the considered organisms in Additional file 1: Figure S4.

**Fig. 4:**
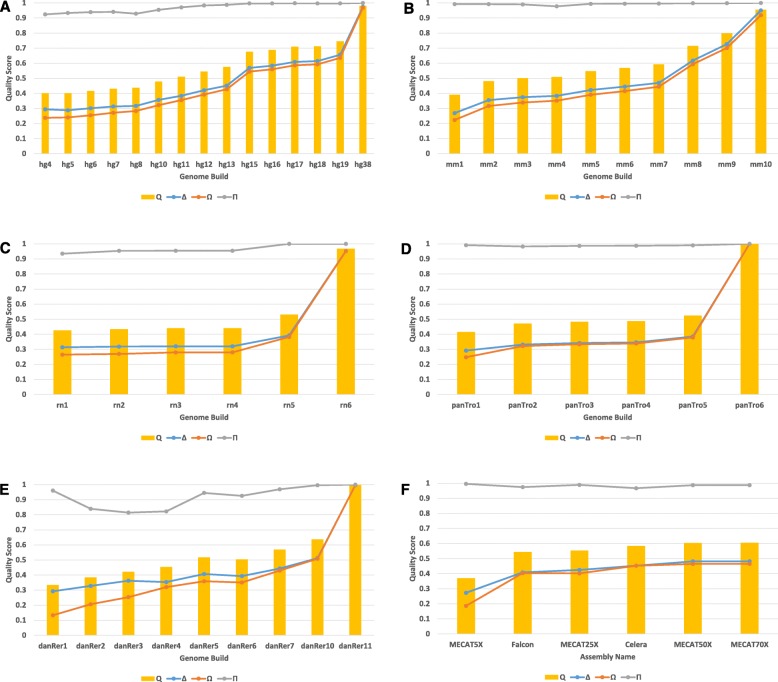
Quality scores for reference genome builds and de novo assemblies of NA24385. The x-axis depicts the builds or assemblies. The y-axis indicates quality scores that dnAQET computed for the reference genome builds of human (**a**), mouse (**b**), rat (**c**), chimpanzee (**d**), zebrafish (**e**) and the de novo assemblies for NA24385 (**f**). The yellow bars show the final quality scores Q. The lines give the area under the cumulative scaffold quality scores distribution curve *Δ* (blue), the area under the cumulative genome coverage curve *Ω* (light brown), and the inverted duplication ratio *Π* (grey)

The final quality scores computed by dnAQET for the reference genome builds for the five organisms and the de novo assemblies of NA24385 were plotted in Fig. 4. The quality scores of the more recent versions of the genome builds were consistently higher than the older builds except one case. The quality score of the zebrafish reference genome build danRer6 had a slightly smaller quality score than the older build danRer5. Examining the quality scores for human reference genome builds (Fig. 4a), we observed that the quality scores almost monotonically increased from 0.401 for hg4 up to 0.575 for hg13. Then the quality score of hg15 jumped up to 0.676 and the quality scores stabilized at around 0.7 for all more recent builds up to hg19, while keeping a slow increase rate. Finally, the quality score reached 0.98 for hg38. The dramatic quality shift between hg13 and hg15, was concurrent with the introduction of the first finished human genome assembly, dated April 2003 [31]. These results clearly showed that the later versions of the human reference genome builds had higher quality scores from dnAQET, which was very consistent with the expectation that the build quality increased with each newly introduced build.

It is interesting to note that dnAQET gave a quality score of 0.98 to hg38 instead of a perfect score of 1, since hg38 was scored against itself. It was not possible to compute a perfect score for hg38 even when it was compared back to itself due to the ambiguous base pairs it contains (5% of the genome) and the misassembly observed due to the repetitive sequences (which may have caused misalignment). Since dnAQET took the artifacts due to alignment tools into consideration, it computed a nearly perfect score for hg38 assembly. It is also important to note that the quality scores of the latest builds for rat, chimpanzee and zebrafish genomes were remarkably higher than those of their predecessor builds. The striking quality shift between the rat builds rn5 and rn6 could be attributed to the introduction of additional PacBio data with 10X coverage into the assembly process [32]. Similarly, the zebrafish reference build danRer11 had a significantly higher quality score than danRer10 due to additional assemblies WGS29 (CAAK00000000.1) and WGS32 (CZQB00000000.1) added where necessary, to fill the gaps [33]. Finally, the quality score for the recently introduced chimpanzee genome build panTro6 was much higher than that of panTro5 because of the high depth of coverage (124X) data used for the assembly and the three-stage progressive assembly methodology which incorporated data from multiple platforms [34].

Since there was no other tool in the literature that could report a single quality score to unify multiple measures for a de novo assembly, we could not directly compare the quality scores that dnAQET computed for these assembly builds with other tools. Consequently, we decided to compare the rankings of the dnAQET quality scores with the rankings from QUAST-LG metrics for these builds. QUAST-LG could provide 37 quality metrics to compare multiple assemblies [22]. Except benchmarking universal single-copy orthologs (BUSCO) completeness and k-mer-based completeness (both metrics require the reads used to generate the assemblies), the remaining 35 QUAST-LG quality metrics were calculated for almost all the reference genome builds (except metrics such as *NA75*, *NG75*, *NGA50*, *NGA75* etc., which could not be computed for some of the earlier builds of zebrafish and mouse). To be consistent in our comparison across multiple organisms, metrics that were not reported for the reference builds of all organisms were discarded, resulting 13 metrics that were reported for all reference genome builds by QUAST-LG and provided a clear ranking of the builds without any ties (see Additional file 2: Tables S6–S10 for detail).

We next computed Pearson correlation coefficients between the rankings provided by each of these quality metrics and the rankings of these builds (the hypothesis for determining the ranking of the builds: the more recent a build is, the better is its quality, and thus a higher rank it has). The result given in Table 1 shows that the rankings of the dnAQET quality scores always had the highest Pearson correlation coefficients and were consistent with the inherent rankings dictated by the hypothesis. These results demonstrated that the dnAQET quality scores reflected the quality of these genome builds better than any of the other metrics due to its sophisticated design to combine different aspects of an assembly into a single value.

**Table 1:** The Pearson correlation coefficients between the rankings of quality scores yielded from dnAQET and QUAST-LG and the rankings of the reference genome builds by hypothesis

	Human	Mouse	Rat	Chimpanzee	Zebrafish
1. dnAQET Score	1.00	1.00	1.00	1.00	0.98
2. Genome fraction (%)	1.00	1.00	1.00	1.00	0.95
3. Total aligned length	0.97	0.99	0.94	0.94	0.75
4. # indels per 100 kbp	1.00	0.95	1.00	0.66	0.98
5. # mismatches per 100 kbp	0.99	0.99	1.00	0.49	0.98
6. NA50	1.00	0.96	1.00	0.43	0.97
7. LA50	1.00	0.96	1.00	0.43	0.97
8. # scaffold gap ext. mis.	0.99	0.96	0.94	0.43	0.97
9. # N’s per 100 kbp	0.89	0.99	0.43	1.00	0.82
10. # misassemblies	1.00	0.96	0.83	0.26	0.90
11. # local misassemblies	0.99	0.99	0.94	0.09	0.88
12. Unaligned length	0.98	0.96	1.00	0.09	0.83
13. Misass. contigs length	0.73	0.41	0.26	0.60	−0.22
14. Total length	−0.64	0.36	0.54	−0.60	0.70

Although the *genome fraction (%)* from QUAST-LG achieved the same performance as the dnAQET quality score in terms of the rankings of genome builds for human, mouse, rat and chimpanzee, dnAQET quality score outperformed the *genome fraction (%)* metric in ranking of the builds of zebrafish genome. Like the *genome fraction (%)* metric, any single metric from QUAST-LG (Table 1) is not able to reliably rank the genome builds compared to dnAQET quality score and thus is not suitable for assessing quality of de novo assemblies as a whole. These metrics focus only on one side of the assemblies and evaluate them by examining from only a single perspective. For instance, the *genome fraction (%)* just reported how much of the reference genome was covered by a de novo assembly without considering the quality of the individual scaffolds. For the reference genome builds, the total *genome fraction (%)* increased almost every time for a more recent build, but this may not be the case for other de novo assemblies. For example, one assembly might have a slightly higher *genome fraction (%)* but lower *N50* (or *NA50*) value and higher *number of misassemblies* than another assembly. On another case, the *genome fraction (%)* of multiple assemblies may indicate a reverse ranking of the assemblies when they are ranked with respect to another metric. Another example that demonstrated the inconsistency of these metrics was the poor performance of metrics such as *number of indels per 100kbp*, *number of mismatches per 100kbp, NA50, LA50* when they were used to rank the chimpanzee genome builds. Although these metrics’ rankings had high Pearson correlation coefficients when used on human, mouse, rat and zebrafish data, they considerably failed in correctly ranking chimpanzee genome builds. Therefore, assessing the quality of an assembly using one metric from QUAST-LG could not provide sufficient information about its overall quality. We see a clear need for a quality metric that unifies these multiple crucial metrics into a single quality value that can be used to reliably assess the quality of an assembly. This need was met by the meticulously designed quality score formulation of dnAQET that united multiple metrics into a single metric.

### Quality of six NA24385 assemblies

The quality score distributions of the contigs of the six assemblies were plotted in in Fig. 3f. None of the six assemblies had any contigs with a quality score higher than 0.8. Both MECAT assemblies generated with 50X and 70X coverage data had contigs with quality scores higher than 0.7 but the percentage of these contigs was only 0.1%. From this perspective, none of the real data assemblies had an outstanding performance in terms of the contig quality scores. It is interesting to note that the best performing assemblies were generated by MECAT on the data of 70X, 50X and 25X coverages. More than 85% of the contigs in these assemblies had quality scores higher than 0.2. In contrast with MECAT, Falcon and Celera assemblies only had 44 and 53% of contigs with quality score higher than 0.2, respectively.

The overall quality scores computed by dnAQET for the six de novo assemblies were presented in Fig. 4f. The assemblies generated by MECAT assembler with high coverage data (50X and 70X) had the best quality score 0.6. The Celera assembly also had a quality score of 0.58, which is slightly lower than the scores of the leading assemblies. The assembly generated by MECAT using 25X coverage had a quality score of 0.55 and followed by the assembly from Falcon with quality score of 0.54. The lowest quality score of 0.37 was obtained from MECAT assembly created with 5X coverage data, which was not surprising because it was generated with the very low coverage data.

We also evaluated these assemblies using QUAST-LG. The computed quality metrics were presented in Additional file 2: Table S11. As mentioned before, some of these metrics produced multiple rankings of these assemblies that contradict with each other. For instance, using *NA50* metric, these assemblies were ranked as MECAT70X, MECAT50X, Falcon, Celera, MECAT25X and MECAT5X in the decreasing order (i.e., higher the *NA50*, better the assembly). However, the ranking was completely changed to Celera, Falcon, MECAT50X, MECAT70X, MECAT25X and finally MECAT5X when *genome fraction (%)* was used as the ranking metric in the decreasing order. When *number of misassemblies* metric was used to order them, the ranking was MECAT5X, MECAT25X, MECAT70X, MECAT50X, Celera and Falcon, in an increasing number of misassemblies (i.e., lower the misassembly, better the assembly). Thus, it was evident from these results that there was a need to consolidate these metrics into a single value, as done by dnAQET, to fairly evaluate the overall quality of these assemblies.

Since there was no inherent ranking of these real data assemblies available to compare the rankings of QUAST-LG metrics with that of dnAQET scores, we used an approach proposed in [18] to infer a reliable ranking that would be considered closest to the ground truth. In that study, the authors calculated the z-scores for each metric for all assemblies in consideration, then summed these scores and finally ranked the assemblies based on the summed z-scores. This was called the *z-score-based ranking*. To obtain the ground truth ranking for our assemblies, we applied a similar approach to 24 metrics, which gave a clear ranking of the assemblies without a tie and were commonly reported for all assemblies, computed by QUAST-LG. We calculated the z-score for each metric and summed the z-scores for each assembly as presented in Additional file 2: Table S11. According to the *z-score-based ranking*, the ranking of these assemblies was MECAT70X, MECAT50X, MECAT25X, Celera, Falcon and MECAT5X in decreasing order (i.e., higher the z-score, better the assembly). The Pearson correlation coefficient between dnAQET quality score ranking of these assemblies and the z-score-based ranking was 0.94 whereas the QUAST-LG metrics, namely *Largest contig*, *N50*, *NG50*, *N75*, *L50*, *LG50*, *L75*, *Largest alignment*, *NGA50* and *NA75* provided the second-high correlation coefficient value, which was 0.77. The results indicated that dnAQET’s quality score outperformed the other metrics based on the *z-score-based ranking*.

### Runtime and peak memory usage

We compared the runtime and memory performances of dnAQET with those of QUAST-LG using the human reference genome builds and the six de novo assemblies of sample NA24385. All the benchmarking tests were done on a server with 125.5 gigabytes of memory running 24 cores of Intel Xeon E5–2680 v3 2.50GHz CPUs. To be able to fairly evaluate the performance of dnAQET against QUAST-LG, we partitioned neither the reference genome nor the input genome builds to be evaluated. Both dnAQET and QUAST-LG were run using 12 threads on a single server and Minimap2 was used as the alignment tool.

**Fig. 5:**
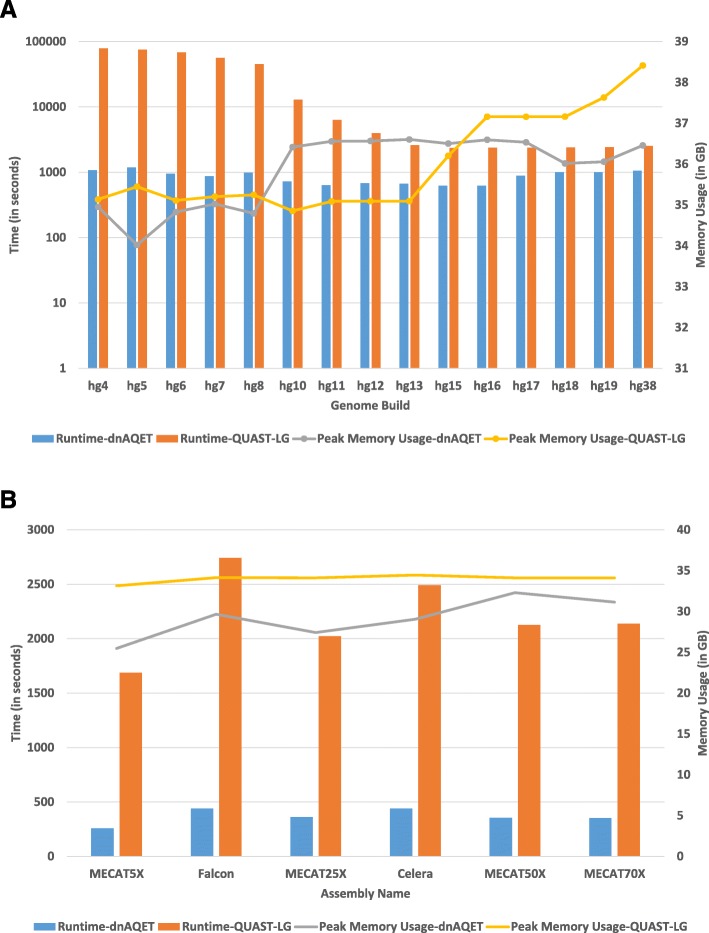
Runtime and peak memory usage**.** The dnAQET (blue bars) and QUAST-LG (orange bars) runtimes to process the human reference genome builds (**a**) and the six assemblies of NA24385 (**b**) were plotted as the bars (left y-axis). The peak memory usages (in gigabytes) were plotted in the lines (right y-axis) for dnAQET (gray lines) and QUAST-LG (yellow lines)

The runtime and peak memory usage of dnAQET and QUAST-LG for the human reference genome builds and the de novo assemblies are presented in Fig. 5. Clearly, the runtime of dnAQET was very stable across all the human reference genome builds and it took 1184 s at the worst case for dnAQET to process a human reference genome build. On the other hand, QUAST-LG performed poorly for the earlier builds (Fig. 5a). For example, QUAST-LG took approximately 21.8 h to finish processing build hg4. QUAST-LG performed better on the more recent builds and its runtime was decreased to approximately 2500 s for the most recent builds. For all six NA24385 assemblies, dnAQET was faster than QUAST-LG to process the assemblies (Fig. 5b). It took 440 s for dnAQET to process the Celera assembly, which was the slowest performance of dnAQET for these assemblies. On the other hand, QUAST-LG’s best runtime was 1686 s for MECAT5X assembly.

In terms of the peak memory usage, both tools used more than 35 gigabytes of memory when analyzing the human reference builds. The dnAQET’s memory usage increased from hg8 to hg10, reaching to 36.5 gigabytes on average, and stabilizing at this number for the later builds. However, QUAST-LG had a steady peak memory usage of 35 gigabytes for builds hg4 to hg13. But starting with hg15, the peak memory usage went up, reaching 38.5 gigabytes for hg38. For NA24385 assemblies, the peak memory usage of dnAQET stayed stable at around 30 gigabytes whereas QUAST-LG’s memory usage was stable at around 35 gigabytes. Our results demonstrated dnAQET outperformed QUAST-LG in terms of execution time; but both tools had comparable peak memory usage.

## Discussion

In the precision medicine era [35], to be able to develop personal treatments, it is of great importance to detect genomic mutations of an individual over the course of life span which is mostly dependent on accurately identifying the background of an individual’s personal genome under its normal state. The current standard method for interrogation of genetic variation using NGS data is to map reads to a trusted reference genome and analyze the alignments. The alternative approach is the de novo assembly of a personal genome. Although in theory the de novo assembly of personal genomes is very promising to be the ideal method for the full discovery of the variation [36], there are still many challenges for it to be used as the primary method for genomic variation detection utilizing the data generated by current NGS technology.

Generating personal genomes at an affordable cost has becoming a more reachable goal as the second and third generation sequencing technologies enabling cost efficient sequencing of individuals and this leads to the development of various de novo genome assembly methods. With the increasing number of tools, it has become a crucial task to devise metrics that could evaluate performance of the de novo assemblies generated by these tools. In this work, we proposed a framework to compute quality measures based on a trusted reference genome to evaluate (i) quality of the scaffolds (or contigs) of a de novo assembly and (ii) the overall quality of a de novo assembly based on the individual qualities of its scaffolds. Our framework combines multiple quality indicators in the quality score calculation, including the length of a scaffold, the total misassembly observed in a scaffold, and the total alignment ratio of a scaffold to the given reference genome. These metrics were carefully amalgamated into a single quality score that assesses quality of the scaffolds with respect to a given reference genome. For calculation of the quality score for an assembly, dnAQET considers multiple metrics including the cumulative reference genome coverage by its scaffolds at different quality thresholds, the cumulative base pair ratio of the scaffolds at different quality thresholds, and the redundancy observed in the assembly. We carefully designed the integration of different quality measurements so that a single quality score hints multiple aspects of assembly quality and measures the overall quality of an assembly.

The individual quality metrics such as *NA50*, *genome fraction (%)*, *number of misassemblies* etc. reported by other tools such as QUAST-LG are not always consistent to indicate the quality of an assembly, sometimes even contradictory with each other when ranking multiple assemblies. We showed that none of these metrics provided coherent results for both the genome reference builds and NA24385 assembly datasets. As an example, we consider the *genome fraction (%)* metric, which performed well for reference genome builds but had a really poor performance for assessing the NA24385 assemblies. This clearly shows it is very challenging for users to select one or several of these quality metrics for comparing multiple assemblies. Individually, each of these metrics represents a different aspect of the quality of a de novo assembly but it is not apparent to users how to utilize the quality metrics obtained. Therefore, it would be much easier for users if a single quality score could consolidate different quality aspects together to assess the quality of an assembly. Our dnAQET quality score fulfills this need by combining multiple crucial metrics into a single score to give an overall snapshot of the quality of assemblies.

Using in-silico datasets, we demonstrated that the dnAQET reported higher quality scores for assemblies with longer scaffolds than the ones with relatively shorter scaffolds. The quality scores of the assemblies with lower number of misassemblies were considerably better than the highly misassembled ones. Furthermore, the assemblies covering a larger portion of the reference genome (up to 1X) obtained higher quality scores than the assemblies with lower reference coverages. Finally, we showed that the dnAQET assigned lower quality scores to more redundant assemblies.

The analysis on the reference genome builds of five organisms demonstrated that the chromosomes in earlier builds of the genome assemblies exhibited lower quality scores than the chromosomes from the later builds. Moreover, we observed the same pattern in terms of the quality scores for the whole assemblies: the earlier builds had always lower quality scores than the more recent builds. When the same reference genome was used to compare two assemblies, dnAQET assigned a higher quality score to the better built assembly, demonstrating that dnAQET is an invaluable benchmarking tool for comparing the quality of assemblies. Furthermore, our framework’s quality score provided the best Pearson correlation coefficients for ranking the genome builds of all five organisms analyzed and the de novo assemblies of NA24385, further indicating that dnAQET quality score scheme reflects the quality of a de novo assembly better than any other metric by combining multiple crucial metrics into a single quality measure.

## Conclusions

The dnAQET is a framework designed to compute quality measures to evaluate quality of both individual scaffolds and a whole de novo assembly. The quality score computed by dnAQET for a scaffold combines multiple quality indicators, including the length of a scaffold, the total misassembly observed in a scaffold, and the total alignment ratio of a scaffold to a given reference genome. For the assembly quality score computation, multiple metrics are integrated into a single robust and reliable quality score, which insinuates multiple aspects of quality of the assembly, hence measuring the overall quality of an assembly. In this respect, dnAQET’s quality score is very convenient to be used as a benchmarking metric when comparing quality of multiple assemblies.

Furthermore, dnAQET is very efficient and scalable for assessing the quality of a de novo genome assembly. To attain the superior performance to its competitors, it partitions both the reference genome and the de novo assembly into smaller parts so that each partition of the assembly can be aligned to the reference genome partition in parallel. Using this strategy, dnAQET achieves a high level of parallelization for the alignment step that enables the tool to scale the quality evaluation processes for large de novo assemblies.

## Methods

The dnAQET is a Java package designed to be used in a Unix based operating system (such as Linux, MacOS, etc.) and it requires Java 1.7 (or a more recent version) Runtime Environment installed. For the alignment step of dnAQET, we used version 2.11 of Minimap2 and 4.0.0 beta2 version of Mummer4 and these versions were included in the dnAQET package. For performance testing, we used 5.0.0rc1 version of QUAST-LG and MECAT V1.3 with their default parameters. Those were the most recent versions of these tools at the time we implemented dnAQET.

## Additional files

Additional file 1: **Figure S1.** The overall flow diagram of dnAQET. **Figure S2.** Visual description of cumulative quality distribution graph and cumulative genome coverage graph. **Figure S3.** Graphical representation of redundancy. **Figure S4.** Individual quality score distributions of the chromosomes of reference genomes for the five organisms across different builds. (PDF 374 kb)

Additional file 2: **Table S1.** The model coefficients for human chrosomomes. The table displays the coefficients for the length, the relocation threshold and the intercept for the linear regression models obtained from the hg38 build of the human genome chromosomes. **Table S2.** The model coefficients for chimpanzee chrosomomes. The table displays the coefficients for the length, the relocation threshold and the intercept for the linear regression models obtained from the panTro6 build of the chimpanzee genome chromosomes. **Table S3.** The model coefficients for mouse chrosomomes. The table displays the coefficients for the length, the relocation threshold and the intercept for the linear regression models obtained from the mm10 build of the mouse genome chromosomes. **Table S4.** The model coefficients for rat chrosomomes. The table displays the coefficients for the length, the relocation threshold and the intercept for the linear regression models obtained from the rn6 build of the rat genome chromosomes. **Table S5.** The model coefficients for zebrafish chrosomomes. The table displays the coefficients for the length, the relocation threshold and the intercept for the linear regression models obtained from the danRer11 build of the zebrafish genome chromosomes. **Table S6.** Computed QUAST-LG metrics for human reference genome builds. **Table S7.** Computed QUAST-LG metrics for mouse reference genome builds. **Table S8.** Computed QUAST-LG metrics for rat reference genome builds. **Table S9.** Computed QUAST-LG metrics for chimpanzee reference genome builds. **Table S10.** Computed QUAST-LG metrics for zebrafish reference genome builds. **Table S11.** Computed QUAST-LG metrics, z-scores of the metrics, the rankings based on the z-scores and finally the Pearson Correlation coefficients of these rankings when compared to the summed z-score based ranking (i.e., the ground truth) for NA24835 assemblies. (XLSX 52 kb)

## Data Availability

The source codes and related documentation for dnAQET are available at https://www.fda.gov/science-research/bioinformatics-tools/de-novo-assembly-quality-evaluation-tool-dnaqet.

## Abbreviations

BUSCO: Benchmarking universal single-copy orthologs
GIAB: Genome in a Bottle
HPC: High-performance computing
NGS: Next generation sequencing
NIST: National Institute of Standards and Technology
UCSC: University of California, Santa Cruz

## References

[1] Xiao Wenming, Wu Leihong, Yavas Gokhan, Simonyan Vahan, Ning Baitang, Hong Huixiao (2016). Challenges, Solutions, and Quality Metrics of Personal Genome Assembly in Advancing Precision Medicine. Pharmaceutics.

[2] Wheeler DA, Srinivasan M, Egholm M, Shen Y, Chen L, McGuire A (2008). The complete genome of an individual by massively parallel DNA sequencing. Nature..

[3] Levy S, Sutton G, Ng PC, Feuk L, Halpern AL, Walenz BP (2007). The diploid genome sequence of an individual human. PLoS Biol.

[4] Wang J, Wang W, Li R, Li Y, Tian G, Goodman L (2008). The diploid genome sequence of an Asian individual. Nature..

[5] Kim JI, Ju YS, Park H, Kim S, Lee S, Yi JH (2009). A highly annotated whole-genome sequence of a Korean individual. Nature..

[6] Li Y, Zheng H, Luo R, Wu H, Zhu H, Li R (2011). Structural variation in two human genomes mapped at single-nucleotide resolution by whole genome de novo assembly. Nat Biotechnol.

[7] Weisenfeld NI, Kumar V, Shah P, Church DM, Jaffe DB (2017). Direct determination of diploid genome sequences. Genome Res.

[8] Luo R, Liu B, Xie Y, Li Z, Huang W, Yuan J (2012). SOAPdenovo2: an empirically improved memory-efficient short-read de novo assembler. Gigascience..

[9] Gnerre S, Maccallum I, Przybylski D, Ribeiro FJ, Burton JN, Walker BJ (2011). High-quality draft assemblies of mammalian genomes from massively parallel sequence data. Proc Natl Acad Sci U S A.

[10] Jackman SD, Vandervalk BP, Mohamadi H, Chu J, Yeo S, Hammond SA (2017). ABySS 2.0: resource-efficient assembly of large genomes using a bloom filter. Genome Res.

[11] Zimin AV, Marçais G, Puiu D, Roberts M, Salzberg SL, Yorke JA (2013). The MaSuRCA genome assembler. Bioinformatics..

[12] Bankevich A, Nurk S, Antipov D, Gurevich AA, Dvorkin M, Kulikov AS (2012). SPAdes: a new genome assembly algorithm and its applications to single-cell sequencing. J Comput Biol.

[13] Koren S, Walenz BP, Berlin K, Miller JR, Bergman NH, Phillippy AM (2017). Canu: scalable and accurate long-read assembly via adaptive k-mer weighting and repeat separation. Genome Res.

[14] Xiao CL, Chen Y, Xie SQ, Chen KN, Wang Y, Han Y (2017). MECAT: fast mapping, error correction, and de novo assembly for single-molecule sequencing reads. Nat Methods.

[15] Miller JR, Delcher AL, Koren S, Venter E, Walenz BP, Brownley A (2008). Aggressive assembly of pyrosequencing reads with mates. Bioinformatics..

[16] Chin CS, Peluso P, Sedlazeck FJ, Nattestad M, Concepcion GT, Clum A (2016). Phased diploid genome assembly with single-molecule real-time sequencing. Nat Methods.

[17] Earl D, Bradnam K, St John J, Darling A, Lin D, Fass J (2011). Assemblathon 1: a competitive assessment of de novo short read assembly methods. Genome Res.

[18] Bradnam KR, Fass JN, Alexandrov A, Baranay P, Bechner M, Birol I (2013). Assemblathon 2: evaluating de novo methods of genome assembly in three vertebrate species. Gigascience..

[19] Salzberg SL, Phillippy AM, Zimin A, Puiu D, Magoc T, Koren S (2012). GAGE: a critical evaluation of genome assemblies and assembly algorithms. Genome Res.

[20] Hunt M, Kikuchi T, Sanders M, Newbold C, Berriman M, Otto TD (2014). REAPR: a universal tool for genome assembly evaluation. Genome Biol.

[21] Gurevich A, Saveliev V, Vyahhi N, Tesler G (2014). QUAST: quality assessment tool for genome assemblies. Bioinformatics..

[22] Mikheenko A, Prjibelski A, Saveliev V, Antipov D, Gurevich A (2018). Versatile genome assembly evaluation with QUAST-LG. Bioinformatics..

[23] Marçais G, Delcher AL, Phillippy AM, Coston R, Salzberg SL, Zimin A (2018). MUMmer4: a fast and versatile genome alignment system. PLoS Comput Biol.

[24] Delcher AL, Salzberg SL, Phillippy AM (2003). Using MUMmer to identify similar regions in large sequence sets. Curr Protoc Bioinformatics.

[25] Li H (2018). Minimap2: pairwise alignment for nucleotide sequences. Bioinformatics..

[26] UCSC Genome Browser Data Download Website. http://hgdownload.soe.ucsc.edu/downloads.html. Accessed 15 Mar 2018.

[27] Genome in a Bottle (GIAB) Website. http://jimb.stanford.edu/giab/. Accessed 10 Mar 2018.

[28] Celera *de novo* Assembly Sequences for Sample NA24385. ftp://ftp-trace.ncbi.nlm.nih.gov/giab/ftp/data/AshkenazimTrio/analysis/UMD_PacBio_Assembly_CA8.3_08252015/trio2.quiver.fasta. Accessed 12 Sep 2018.

[29] Falcon *de novo* Assembly Sequences for Sample NA24385. ftp://ftp-trace.ncbi.nlm.nih.gov/giab/ftp/data/AshkenazimTrio/analysis/MtSinai_PacBio_Assembly_falcon_03282016/hg002_p_and_a_ctg.fa. Accessed 12 Sep 2018.

[30] PacBio Sequencing Data Download Page for Sample NA24385. ftp://ftp-trace.ncbi.nlm.nih.gov/giab/ftp/data/AshkenazimTrio/HG002_NA24385_son/PacBio_MtSinai_NIST/PacBio_fasta/. Accessed 27 Sep 2018.

[31] The First Finished Human Genome Assembly UCSC Webpage. http://hgdownload.soe.ucsc.edu/goldenPath/10april2003/bigZips/. Accessed 29 Oct 2018.

[32] NCBI Assembly Database Webpage for July 2014 (RGSC 6.0/rn6) Assembly of the Rat Genome. https://www.ncbi.nlm.nih.gov/assembly/GCF_000001895.5/. Accessed 29 Oct 2018.

[33] NCBI Assembly Database Webpage for the May 2017 (GRCz11/danRer11) Assembly of the Zebrafish Genome. https://www.ncbi.nlm.nih.gov/assembly/GCF_000002035.6/. Accessed 29 Oct 2018.

[34] NCBI Assembly Database Webpage for the January 2018 (Clint_PTRv2/panTro6) Assembly of the Chimp Genome. https://www.ncbi.nlm.nih.gov/assembly/GCF_002880755.1/. Accessed 29 Oct 2018.

[35] Collins FS, Varmus H (2015). A new initiative on precision medicine. N Engl J Med.

[36] Chaisson MJ, Wilson RK, Eichler EE (2015). Genetic variation and the de novo assembly of human genomes. Nat Rev Genet.

