# Supplementary material for: dnAQET: a framework to compute a consolidated metric for benchmarking quality of de novo assemblies
Source: BMC Genomics. 2019 Sep 11;20:706. doi: 10.1186/s12864-019-6070-x (PMC6737619; doi:10.1186/s12864-019-6070-x)
Supplement: Supplementary file 1 — Figure S1. The overall flow diagram of dnAQET. Figure S2. Visual description of cumulative quality distribution graph and cumulative genome coverage graph. Figure S3. Graphical representation of redundancy. Figure S4. Individual quality score distributions of the chromosomes of reference genomes for the five organisms across different builds. (PDF 374 kb) [file 12864_2019_6070_MOESM1_ESM.pdf]

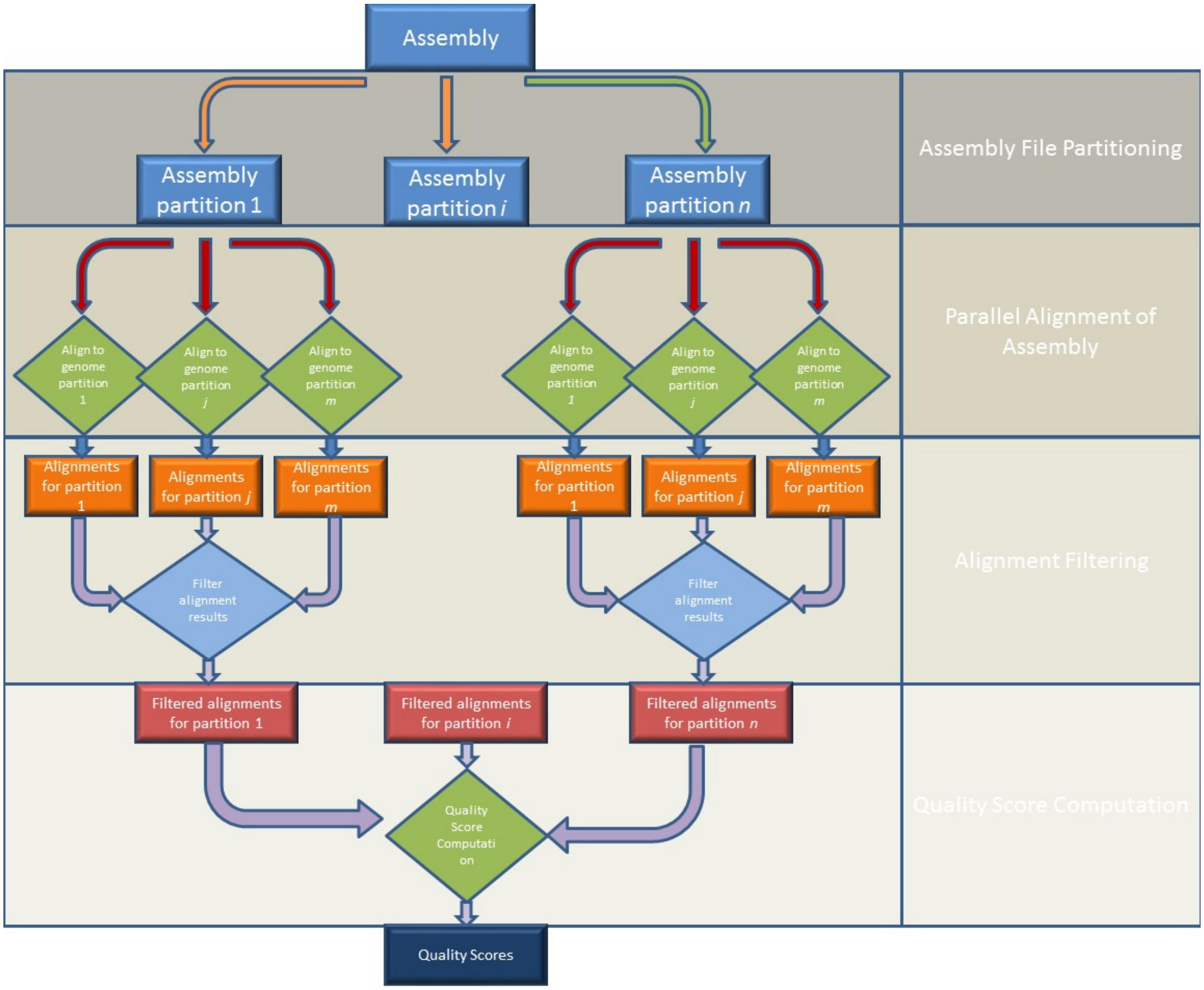

Figure S1. The overall flow diagram of dnAQET. The dnAQET aligns the partitioned assembly in parallel to a partitioned genome. Resulting alignments are filtered and quality score is computed.

A

B

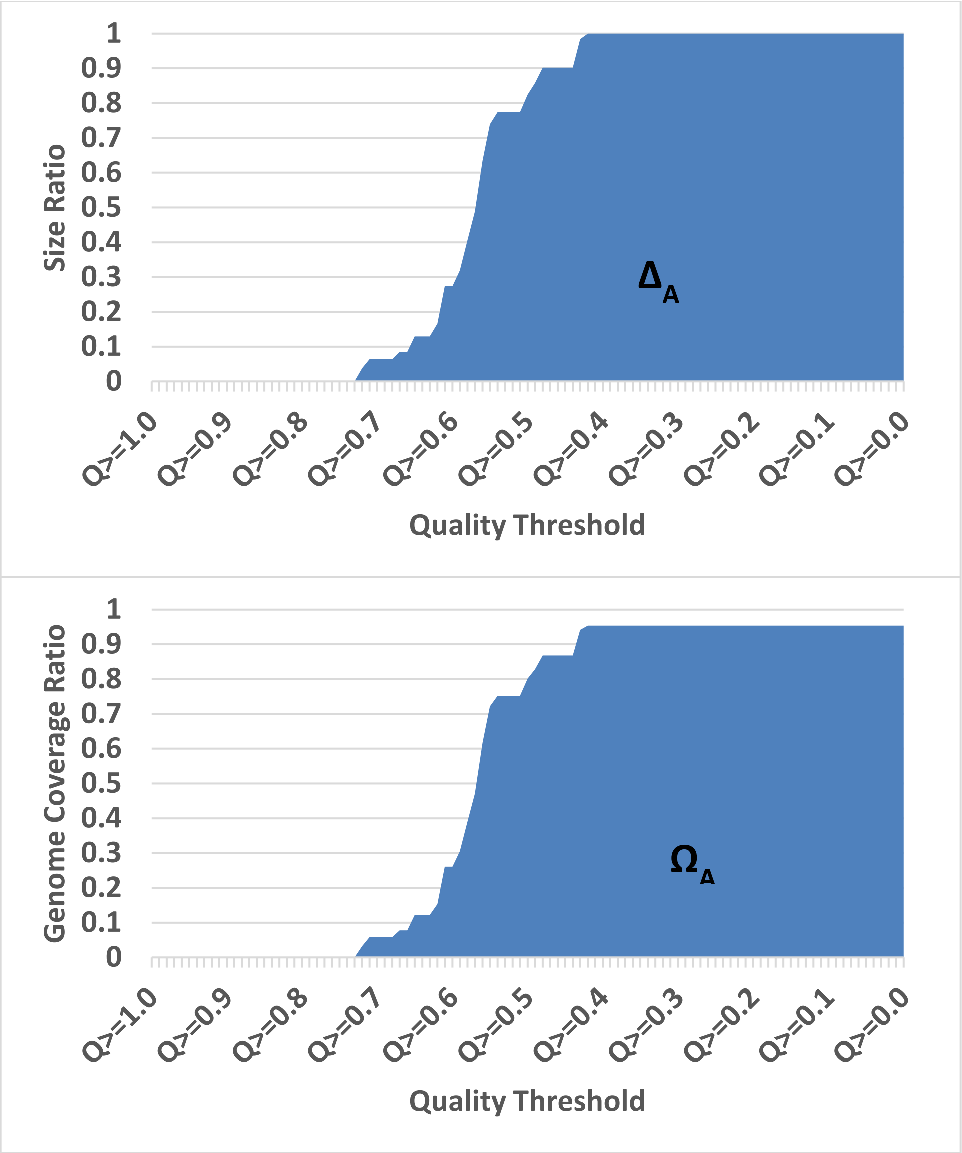

Figure S2. Visual description of cumulative quality distribution graph and cumulative genome coverage graph. The graph in (A) displays a cumulative quality distribution curve for which the values on x-axis represents the quality thresholds and the corresponding values on the y-axis represents the ratio of the total size of the scaffolds that has a quality score more than the corresponding quality threshold on the x-axis to the whole assembly size. Similarly, the graph in (B) a curve for which the values on x-axis represents the quality thresholds and the corresponding values on the y-axis represents the reference genome coverage ratio obtained by the scaffolds that has a quality score more than the corresponding quality threshold on the x-axis.

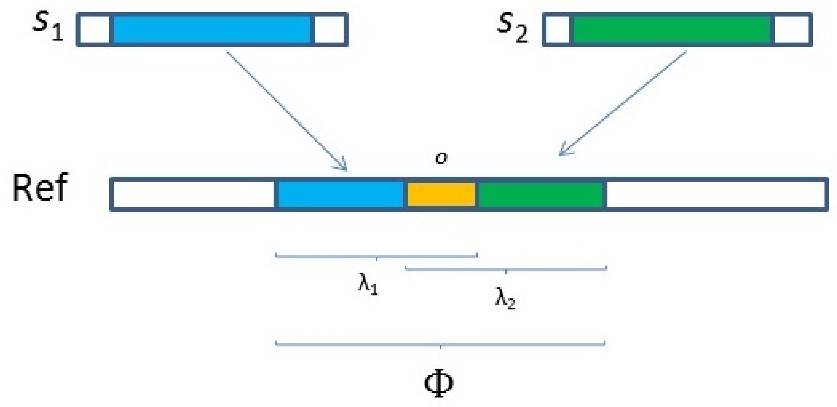

Figure S3. Graphical representation of redundancy. The graph displays two scaffolds with an overlap of *o* base pairs in their alignments. The portion of the reference genome covered by these alignments is denoted by Φ and the alignment lengths of s_1 and s_2 are denoted by λ_1 and λ_2, respectively.

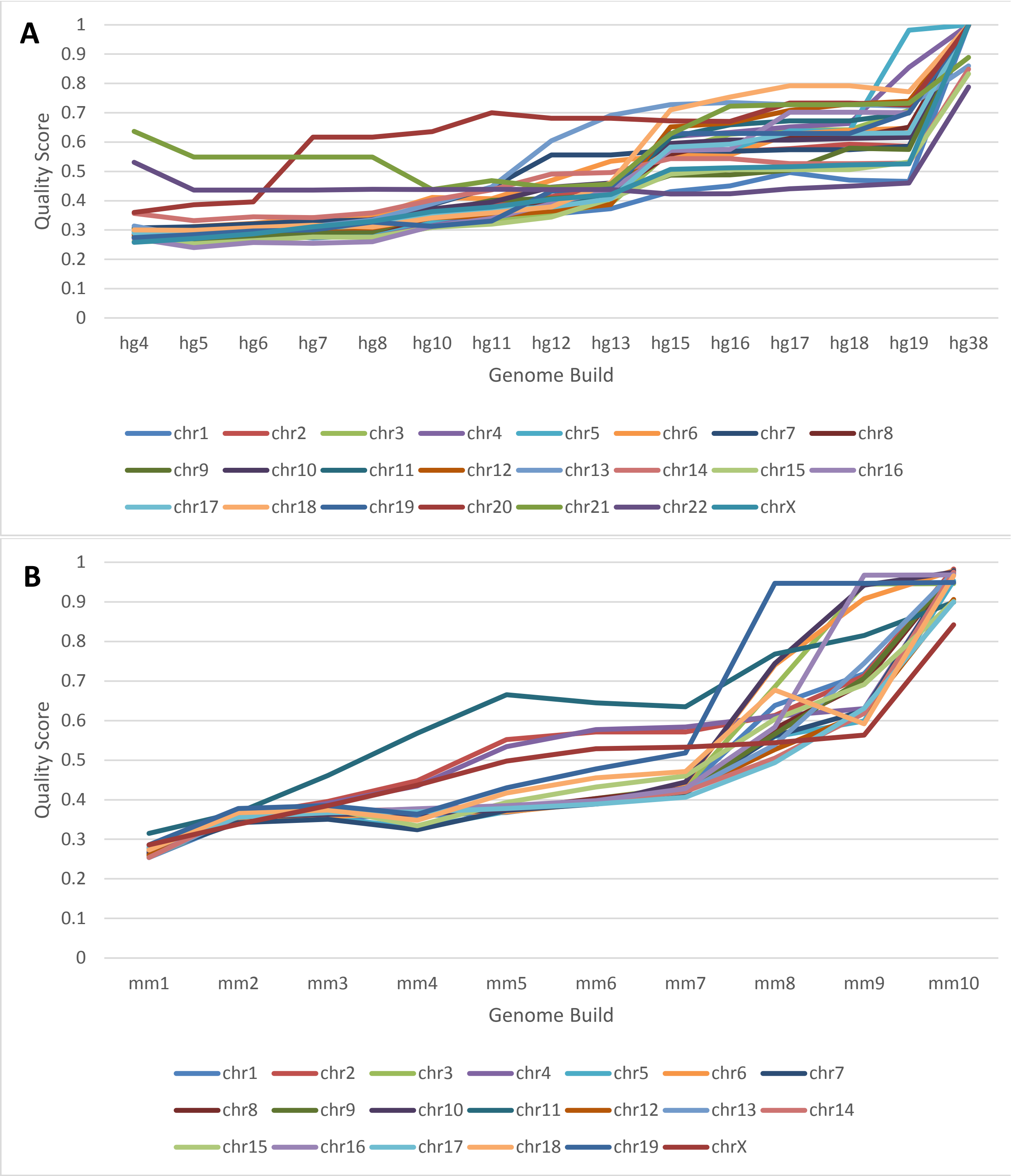
A
B
Quality Score
Genome Build
0
0.1
0.2
0.3
0.4
0.5
0.6
0.7
0.8
0.9
1
hg4
hg5
hg6
hg7
hg8
hg10
hg11
hg12
hg13
hg15
hg16
hg17
hg18
hg19
hg38
mm1
mm2
mm3
mm4
mm5
mm6
mm7
mm8
mm9
mm10
chr1
chr2
chr3
chr4
chr5
chr6
chr7
chr8
chr9
chr10
chr11
chr12
chr13
chr14
chr15
chr16
chr17
chr18
chr19
chr20
chr21
chr22
chrX

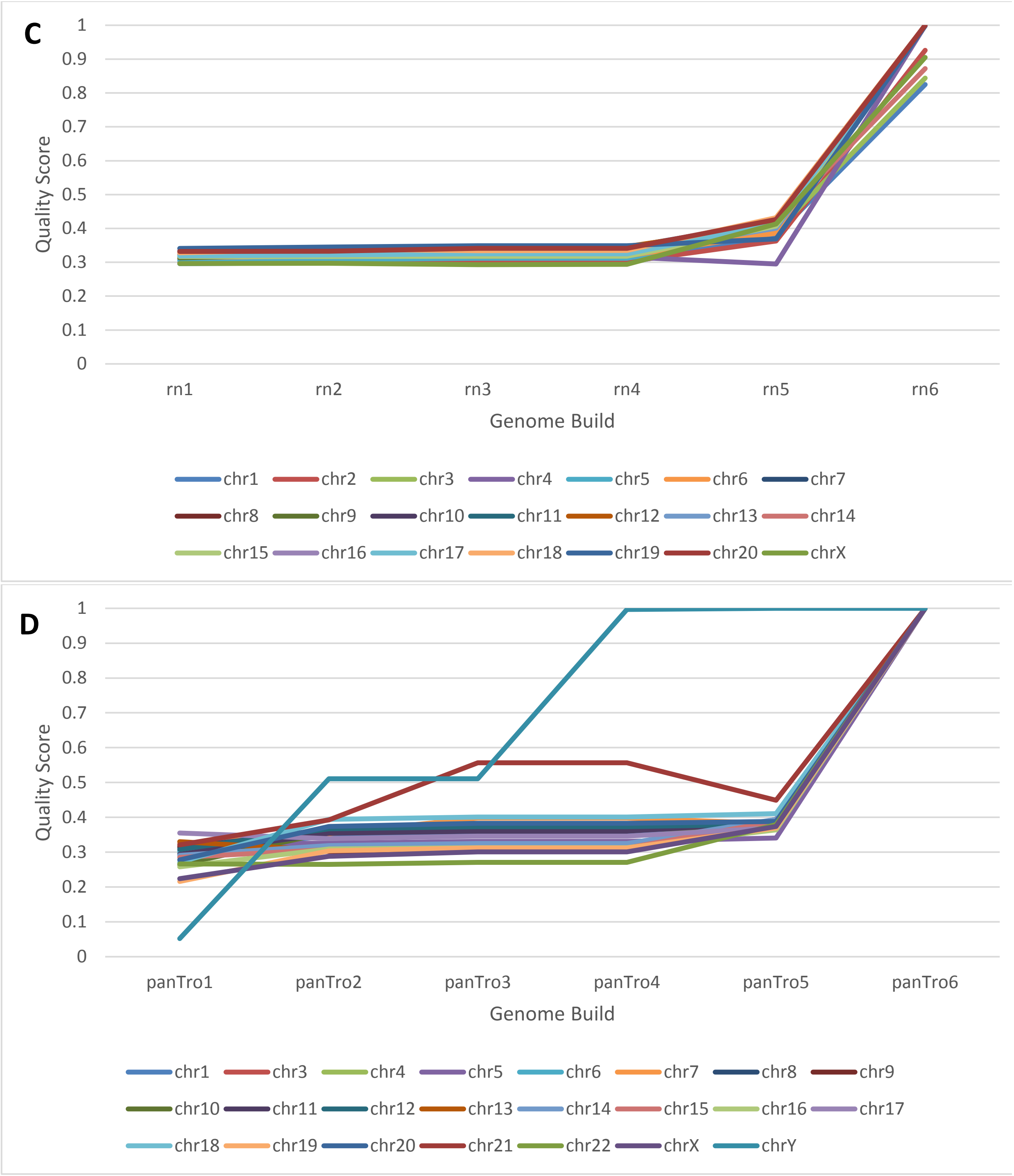

C
Quality Score
1
0.9
0.8
0.7
0.6
0.5
0.4
0.3
0.2
0.1
0
rn1
rn2
rn3
rn4
rn5
rn6
Genome Build
chr1 chr2 chr3 chr4 chr5 chr6 chr7
chr8 chr9 chr10 chr11 chr12 chr13 chr14
chr15 chr16 chr17 chr18 chr19 chr20 chrX
D
Quality Score
1
0.9
0.8
0.7
0.6
0.5
0.4
0.3
0.2
0.1
0
panTro1
panTro2
panTro3
panTro4
panTro5
panTro6
Genome Build
chr1 chr3 chr4 chr5 chr6 chr7 chr8 chr9
chr10 chr11 chr12 chr13 chr14 chr15 chr16 chr17
chr18 chr19 chr20 chr21 chr22 chrX chrY

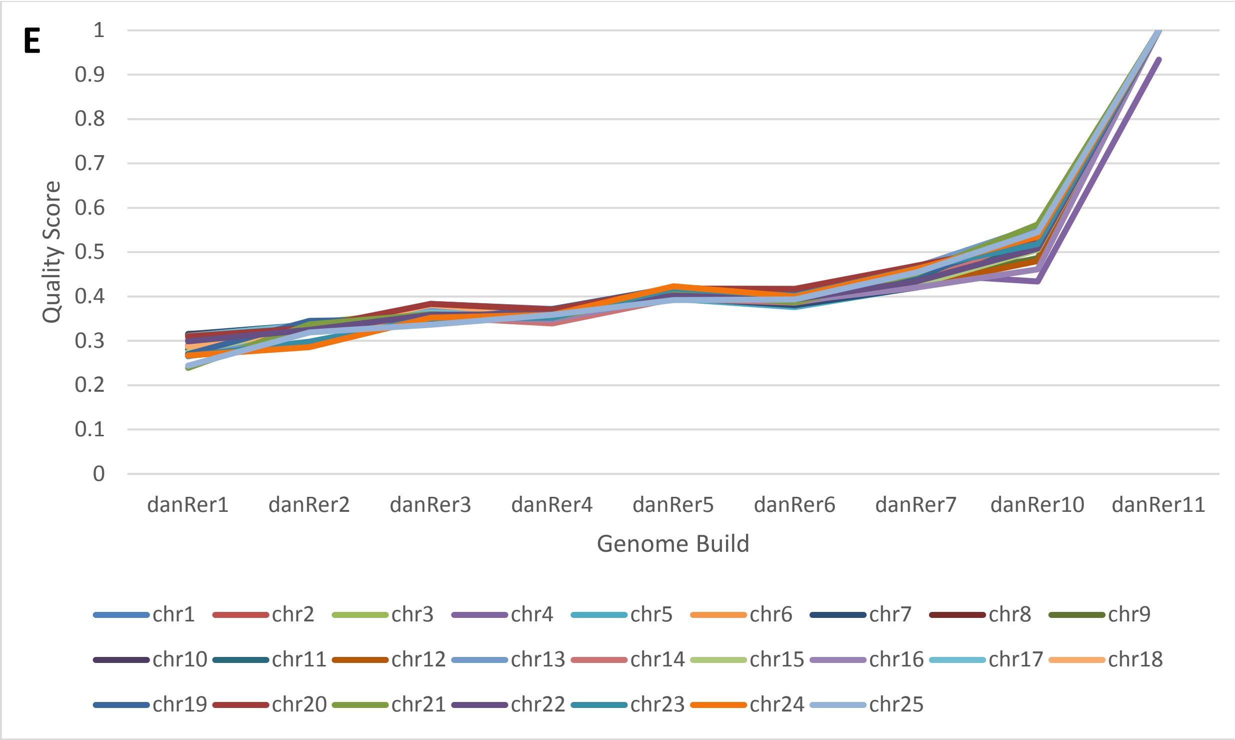

Figure S4. Individual quality score distributions of the chromosomes of reference genomes

for the five organisms across different builds. The x-axis depicts the builds. The y-axis indicates quality scores that dnAQET computed for the chromosomes of reference genome builds of human (A), mouse (B), rat (C), chimpanzee (D), zebrafish (E).
